# Unusual Unsatisfactory Treatment in Two Patients with Imported Cutaneous Leishmaniasis

**DOI:** 10.3390/tropicalmed9100227

**Published:** 2024-09-30

**Authors:** Anna Kuna, Romuald Olszański, Beata Szostakowska, Natalia Kulawiak, Ravi Kant, Maciej Grzybek

**Affiliations:** 1 Department of Tropical and Parasitic Diseases, Faculty of Health Science, Medical University of Gdansk, 81-519 Gdansk, Poland; natalia.kulawiak@gumed.edu.pl (N.K.); ravi.kant@helsinki.fi (R.K.); 2 Specialized Dermatology Outpatient Clinic, 81-366 Gdynia, Poland; romuald.olszanski@wp.pl; 3 Department of Tropical Parasitology, Faculty of Health Science, Medical University of Gdansk, 81-519 Gdansk, Poland; beata.szostakowska@gumed.edu.pl (B.S.); maciej.grzybek@gumed.edu.pl (M.G.); 4 Department of Virology, Helsinki University, 00290 Helsinki, Finland

**Keywords:** leishmaniasis, skin ulcer, tropic, alternative treatment, Kambo cleansing

## Abstract

Cutaneous leishmaniasis is one of the most commonly diagnosed dermatological condition in travel medicine after diarrhoeal diseases and febrile status. The disease is transmitted by *Phlebotomus* and *Lutzomyia* sandflies. It appears in various clinical forms, the most common of which is a painless ulcer with raised edges, usually present on exposed parts of the body on the side where the insect bite occurred. Annually, over a million new cutaneous leishmaniasis (CL) cases are reported globally. We present two cases of affliction, the first occurring in Patient 1, who attempted treatment through the Kambo cleanse in South America, which is considered a toxic, even life-threatening, procedure. It involves the subcutaneous application of a substance dangerous to humans derived from the surface mucus of a frog. Patient 2 applied caustic ointments, a fruitarian diet, and hyperbaric oxygen therapy in a private setting. After initial therapeutic failures caused by the patients’ unconventional treatment ideas, the causal treatment effect was satisfactory, demonstrating the efficacy of these treatments in resolving the infection when applied appropriately. Despite the typical CL presentation in both patients, their self-treatment course was unusual. It is worth noting that alternative, sometimes harmful, self-treatment initiatives by patients may be surprising and ineffective. Promoting knowledge about tropical diseases among travellers and medical staff is crucial to improving treatment outcomes.

## 1. Introduction

Leishmaniasis is a neglected tropical disease prevalent in 90 countries worldwide and found on every continent except Australia and Antarctica [1]. According to data from the Centers for Disease Control and Prevention and the Geosentinell Surveillance Network, skin conditions rank third among ailments presented by travellers, following diarrhoea and febrile illnesses [2,3]. Leishmaniasis is caused by more than 20 species of *Leishmania*, which are transmitted by phlebotomine sandflies belonging to the genus *Phlebotomus* and *Lutzomyia*. Annually, over a million new cutaneous leishmaniasis (CL) cases are reported globally [4]. About 95% of CL cases occur in the Americas, the Mediterranean basin, the Middle East, and Central Asia, with an estimated 600,000 to 1,000,000 new cases occurring worldwide annually, though only around 200,000 are reported to the WHO [5]. Patients in non-endemic countries for tropical diseases often encounter difficulties accessing diagnostic services and appropriate treatment due to medical personnel’s lack of knowledge and experience, the rare occurrence of exotic diseases, and the unavailability of diagnostic methods and causal medications [6]. Patients from tropical, subtropical, or southern European regions often present the classic form of CL, characterised by painless ulcerations on exposed body parts without systemic symptoms [7].

The infections remain unresponsive to empirical systemic and local treatments, including antibiotic therapy or popular ointments and creams. Leishmaniasis is classified as a neglected tropical disease, a diverse group of illnesses caused by various pathogens. Their common characteristics include occurrence in developing countries, pathological health impacts, and negative socio-economic consequences [4,8]. Unfortunately, many of these diseases cannot be prevented through specific prophylaxis, and often, the medications available are outdated and toxic [9]. We describe a case of cutaneous leishmaniasis in a patient who returned from Peru and another case in a patient who returned from Costa Rica and the Dominican Republic. Both presented with the classic form of leishmaniasis; however, their belief in alternative treatment methods and lack of appropriate medical knowledge resulted in an unusual course of the disease in each case. The patients were admitted to the University Centre for Marine and Tropical Medicine: the first patient in 2011 and the second patient in 2023. The work was conducted with the approval of the Independent Bioethics Committee at Gdańsk Medical University.

## 2. Clinical Case Description

**Figure 1:**
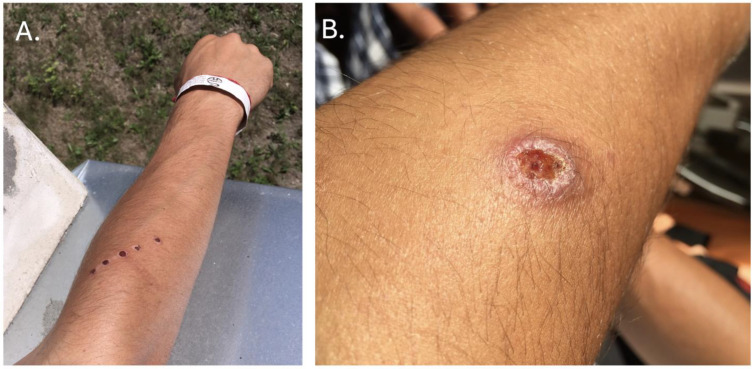
(**A**) Dermatologic issues after Kambo cleansing. (**B**) Typical presentation of CL in Patient 1.

Patient 1. The first patient, a 26-year-old man who had spent several months in Central and South America, primarily in Peru, embraced adventurous travel, closely interacted with local communities, and treated his journey as a spiritual quest. He developed a skin lesion on his left upper limb. He chose to undergo treatment in the Amazon jungle, which was administered by a shaman using the toxic secretion of the Kambo frog. The shaman’s procedure was applied to a healthy area of the body, specifically the right upper limb. The Kambo ceremony involves the application of an extremely toxic secretion from the frog’s skin to the patient via subcutaneous administration using a sharp instrument. Many believe this described “therapy” method can cleanse their mind and body of various ailments. After returning to Poland, he sought medical attention, primarily for persistent, unhealed lesions resulting from applying the frog’s secretions. Only a physical examination revealed an ulceration on the forearm. Figure 1A shows dermatologic issues following Kambo cleansing on the right forearm with a photograph taken upon hospital admission, and Figure 1B depicts the typical presentation of cutaneous leishmaniasis in Patient 1 on the left forearm with a photograph taken before the initiation of the causal treatment. Both images were taken upon hospital admission. The lesion was located on the posterior part of the forearm.

Tissue biopsy samples were collected for microscopic and molecular examination to identify the aetiological agents. The material for examination was collected as a smear on a glass slide, along with a biopsy from the edge of the ulcer and material from the base. Microscopic examination confirmed the presence of amastigote forms. Molecular analysis was performed as follows: DNA was isolated using a commercial kit (DNA Genomic Mini AX Tissue, A&A Biotechnology, Gdańsk, Poland) according to the manufacturer’s instructions. Polymerase Chain Reaction (PCR) was conducted using the primers LGITSF2 (GCATGCC ATATTCTCAGTGTC) and LGITSR2 (GGCCAACGCGAAGTTGAATTC) [7]. The reaction mixture consisted of 12.5 μL of PCR Master MixPlus High GC (a ready-to-use PCR mixture containing Taq DNA polymerase, PCR buffer, MgCl2, and dNTPs; A&A Biotechnology, Gdańsk, Poland), 1 μL of each primer (at a concentration of 10 μM), and 4 μL of DNA template, supplemented with deionised water to a final volume of 25 μL. The PCR reaction conditions were as follows: 5 min at 95 °C (initial denaturation) followed by 40 cycles of 30 s at 95 °C, 30 s at 60 °C, 1 min at 72 °C, and a final extension step of 7 min at 72 °C [10,11,12]. The molecular test confirmed cutaneous leishmaniasis caused by *Leishmania* sp. However, insufficient material did not allow for species identification through sequencing. The intravenous pentamidine isethionate treatment (sterile pentamidine isethionate, 300 mg/vial, white crystalline powder soluble in water) was initiated at 2 mg/kg and lasted seven days. Pentamidine was administered daily at a total dose of 14 mg/kg. This resulted in a gradual improvement, which began after a week of daily pentamidine administration, and healing of the primary lesion was clinically confirmed during the patient’s follow-up visit one month after causal treatment. Additionally, the wounds from the Kambo frog healing ritual closed over time, bringing considerable relief and satisfaction to the patient. The wounds from the cleansing ceremony healed after applying a topical antibiotic containing mupirocin.

**Figure 2:**
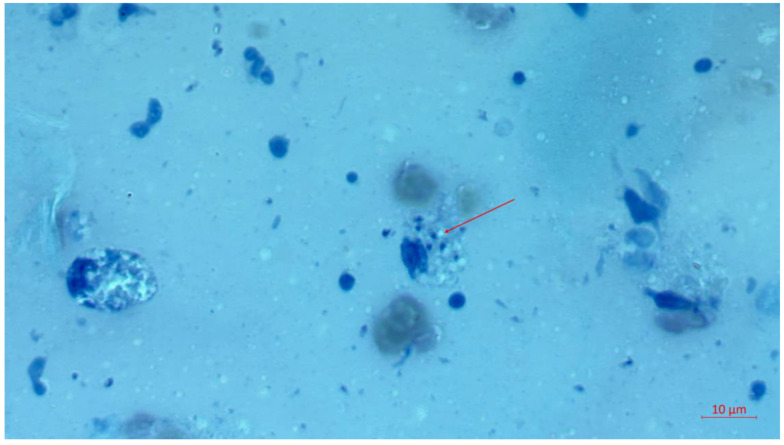
Leishmania amastigotes in a Giemsa-stained tissue scraping. The red arrow indicates amastigotes within a macrophage.

**Figure 3:**
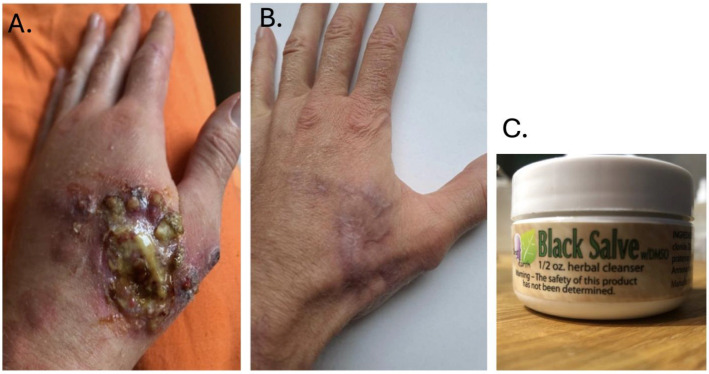
(**A**) Skin changes in Patient 2 before admission to the hospital. (**B**) The effect of antimonial therapy. (**C**) The caustic herbal cream used by Patient 2.

Patient 2. After returning from a four-month journey through Costa Rica and the Dominican Republic, a 40-year-old man (Patient 2) noticed an abrasion on the dorsal surface of his left hand. The lesion appeared five weeks after returning to the country, when the patient began seeking medical assistance. This abrasion progressed into an ulceration. Over the following months, Patient 2 engaged in self-prescribed treatments that included ozone sauna sessions, a mono-diet (initially consisting solely of orange juice, later switching to grapes), consumption of herbal tinctures purchased online as a part of the cleansing regimen, and the application of “Black Salve” ointment recommended by herbalists in Costa Rica. Following the self-initiated treatments, the skin lesion on the dorsal surface of Patient 2’s hand worsened, expanding to cover half its surface and causing the emergence of satellite lesions. Subsequently, he sought professional medical care. The material for examination was collected as a smear on a glass slide, along with a biopsy from the edge of the ulcer and material from the base. Microscopic examination revealed the presence of amastigote forms of *Leishmania* sp. (Figure 2), and molecular test (approach as described above) led to the diagnosis of *Leishmania panamensis* infection (sequence deposited in GenBank under accession number PP952307). The patient underwent a 20-day course of Glucantime, complemented by cryotherapy and hyperbaric oxygen therapy, under the direction of a dermatologist specialising in the treatment of skin diseases acquired in tropical countries. In contrast, the patient privately underwent sessions in a hyperbaric chamber without informing the attending physicians. The meglumine antimoniate (Glucantime) dose was 20 mg/kg/day for 20 days IM. This treatment regimen resulted in slow healing of the ulceration, leaving behind a scar. The patient was pleased with the therapy’s outcome and planned to get an exotic tattoo over the scarred area. The extensive skin lesion in the course of cutaneous leishmaniasis in Patient 2 is shown in Figure 3, which was taken after applying the cream and before initiating causal treatment with antimonials.

## 3. Discussion

Skin lesions following stays in tropical and subtropical regions are a common clinical issue among tourists, second only to traveller’s diarrhoea and feverish states [13]. Cutaneous leishmaniasis is a tropical dermatosis that typically presents as a painless ulceration on exposed body parts and grows slowly. The appearance of skin lesions during infection can be varied, especially in immunocompromised patients, depending on the specific species of the parasite [14]. Prevention of CL includes avoiding insect bites through effective repellents, insecticide-treated bed nets, and protective clothing. Currently, there are no effective chemoprophylaxes or vaccines available for CL. The infection remains unresponsive to empirical systemic and local treatments, including antibiotic therapy or corticosteroids, a characteristic feature of this disease that can further aid in diagnosis. Diagnosis requires parasitological examination of lesion smears, histopathological examination of biopsy specimens, and molecular analysis (PCR) of lesion tissues. It is worth noting that molecular diagnostics are extremely useful in cases where amastigotes are not detected in microscopic examination, yet clinical suspicion of cutaneous leishmaniasis is evident. In addition, sequencing of PCR products allows for the identification of the *Leishmania* species, which is useful for selecting the optimal therapy. Some patients are even diagnosed by oncologists concerned about the clinical appearance of skin lesions suggestive of basal cell carcinoma. Others undergo diagnostics for granulomatous diseases, and many seek help from various specialists before finding a clinician familiar with tropical and parasitic diseases [15]. Effective treatment for CL includes cryotherapy, thermotherapy, antimonials, azoles, and miltefosine. In Poland, most medications used to treat tropical diseases are neither registered nor available in pharmacies. Medications are imported through a government-targeted import program. The form of antimonials available at the hospital pharmacy for the patients was described.

Kambo, a natural substance derived from the glandular secretions of the amphibian *Phyllomedusa bicolor*, is used transdermally in South American rituals for religious purifying purposes. It is also known that applying the toxic substance from this frog can result in side-effects that threaten health and life [16]. In summary, it can be concluded that Patient 1 survived despite undergoing risky self-treatment. The use of alternative treatment methods, rather than pursuing an accurate diagnosis and causal treatment, complicated the disease’s progression and posed a potentially life-threatening risk to the patient. Patient 1 underwent a hazardous therapy involving the application of the frog’s secretion, which may contain several active peptides, including phyllocaeruleins (with hypotensive properties), tachykinins and phyllokinins (vasodilators), dermorphins and deltorphins (with opiate-like actions), and adenoregulins (with antibiotic properties). Additional complications associated with this treatment include hepatitis, hyponatremia, and psychosis [17]. The second patient applied a caustic cream that exacerbated the skin lesions caused by leishmaniasis and struggled to adhere to the hospital treatment regimen. Both patients were previously healthy, with no history of chronic illness, and screening for diseases associated with immunosuppression was negative. As depicted in Figure 3A, the caustic cream likely caused a bacterial superinfection with massive purulent discharge, which may have compromised the quality of the material collected for analysis. It is important to remember that conventional treatment for leishmaniasis is also associated with side-effects. For pentamidine isethionate, nephrotoxicity, hepatotoxicity, pancreatitis leading to insulin-dependent diabetes, hypertension, hypoglycaemia, QT prolongation, hyperkalemia, and vertigo have been reported. Glucantime can cause numerous adverse effects, the most frequent of which include cardiotoxicity, elevated liver function test values, increased urea and creatinine levels, anorexia, nausea, vomiting, myalgia, and arthralgia. When applied intralesionally, it may cause local irritation, pain, oedema, erythema, or pruritus [18,19]. Leishmaniasis is a neglected tropical disease, and as such, there is a lack of effective preventive measures and modern, non-toxic treatment options [20].

In the patients described, testing for leishmaniasis was performed only once. The outcome of the treatment was assessed based on clinical presentation. Therefore, data on a parasitological cure is not available. In both cases, the results of conventional treatments were evaluated during a follow-up hospitalisation, to which both patients were invited after one month. Patient 2 continued to be monitored by a specialist dermatology clinic, while Patient 1 did not return for further evaluation. In addition to the therapeutic challenges dependent on the specific personalities of both patients, in non-endemic countries, diagnosing tropical diseases, including leishmaniasis, poses diagnostic challenges, as many doctors are unfamiliar with this field of medicine [21]. Additionally, treatment can be challenging to access and burdensome for patients. Therefore, improving knowledge of travel and tropical medicine is essential [22], as it also underscores the importance of addressing CL as a neglected disease and advocating for the introduction of modern leishmaniasis treatments.

## 4. Conclusions

Considering changing climatic conditions and increased human migration, knowledge of tropical diseases is becoming increasingly crucial due to the risk of their importation from hot climate countries. Rapid diagnosis and the initiation of causal treatments (e.g., in malaria) are essential in many cases. In others, such as leishmaniasis, empirical therapy with popular preparations used for skin lesions in patients from non-endemic countries is ineffective and can lead to disease progression.

## Data Availability

All data are presented within the manuscript. Obtained sequences were deposited to GenBank and are available under the number PP952307.
